# SARS-CoV-2 Variant Tracking and Mitigation During In-Person Learning at a Midwestern University in the 2020-2021 School Year

**DOI:** 10.1001/jamanetworkopen.2021.46805

**Published:** 2022-02-03

**Authors:** Carolina Avendano, Aaron Lilienfeld, Liz Rulli, Melissa Stephens, Wendy Alvarez Barrios, Joseph Sarro, Michael E. Pfrender, Marie Lynn Miranda

**Affiliations:** 1 Children’s Environmental Health Initiative, University of Notre Dame, Notre Dame, Indiana; 2 Notre Dame Research, University of Notre Dame, Notre Dame, Indiana; 3 Genomics and Bioinformatics Core Facility, University of Notre Dame, Notre Dame, Indiana; 4 Department of Biological Sciences, University of Notre Dame, Notre Dame, Indiana; 5 Department of Applied and Computational Mathematics and Statistics, University of Notre Dame, Notre Dame, Indiana; 6 Department of Pediatrics, Duke University, Durham, North Carolina

## Abstract

**Importance:**

The COVID-19 pandemic led many higher education institutions to close campuses during the 2020-2021 academic year. As campuses prepared for a return to in-person education, many institutions were mandating vaccines for students and considering the same for faculty and staff.

**Objective:**

To determine the association between vaccination coverage and the levels and spread of SARS-CoV-2, even in the presence of highly-transmissible variants and congregate living, at a midsized university in the US.

**Design, Setting, and Participants:**

This case series was conducted at a midsized Midwestern university during the spring 2021 semester. The university developed a saliva-based surveillance program capable of high-throughput SARS-CoV-2 polymerase chain reaction testing and genomic sequencing with the capacity to deliver results in less than 24 hours. On April 7, 2021, the university announced a vaccine requirement for all students for the fall 2021 semester and announced the same requirement for faculty and staff on May 20, 2021. The university hosted an onsite mass vaccination clinic using the 2-dose Pfizer-BioNTech vaccine during April 8 to 15 and April 29 to May 6, 2021. Data were analyzed for 14 894 individuals from the university population who were tested for COVID-19 on campus from January 6 to May 20, 2021.

**Main Outcomes and Measures:**

Positive SARS-CoV-2 diagnosis was confirmed by quantitative reverse transcription–polymerase chain reaction of saliva specimens, and variant identity was assessed by quantitative reverse transcription–polymerase chain reaction and next-generation sequencing of viral genomes.

**Results:**

Between January 6 and May 20, 2021, the university conducted 196 185 COVID-19 tests for 14 894 individuals and identified 1603 positive cases. Within those positive cases, 950 individuals (59.3%) were male, 644 (40.2%) were female, 1426 (89.0%) were students, and 1265 (78.9%) were aged 17 to 22 years. Among the 1603 positive cases, 687 were identified via polymerase chain reaction of saliva specimens. The Alpha (B.1.1.7) variant constituted 218 of the 446 total positives sequenced (48.9%). By May 20, 2021, 10 068 of 11 091 students (90.8%), 814 of 883 faculty (92.2%), and 2081 of 2890 staff (72.0%) were vaccinated. The 7-day rolling average of positive cases peaked at 37 cases on February 17 but declined to zero by May 14, 2021. The 7-day moving average of positive cases was inversely associated with cumulative vaccination coverage, with a statistically significant Pearson correlation coefficient of −0.57 (95% CI, −0.68 to −0.44).

**Conclusions and Relevance:**

This case series study elucidated the association of a robust vaccination program with a statistically significant decrease in positive COVID-19 cases among the study population even in the presence of highly transmissible variants and congregate living.

## Key Points

**Question:**

Is the spread of COVID-19 on a college campus, even in the context of new and highly transmissible variants, negatively associated with vaccination coverage?

**Findings:**

This case series leveraged more than 190 000 COVID-19 surveillance tests for 14 894 individuals, including 1603 positive test results, at a midsized Midwestern university from January 6 to May 20, 2021. By April 2021, the highly transmissible Alpha (B.1.1.7) variant was the only variant resulting in persistent numbers of positive cases. An increase in vaccination coverage was associated with a decrease in COVID-19 cases in the campus population.

**Meaning:**

Mass vaccination efforts were associated with a statistically significant decrease in the spread of SARS-CoV-2 even as highly transmissible variants were introduced in a residential campus setting.

## Teaser

This case series examines the association between vaccine coverage and the spread of SARS-CoV-2 variants at a midsized university in the midwestern US.

## Introduction

After initial nearly universal shutdowns of in-person learning at US colleges and universities at the start of the COVID-19 pandemic in March 2020, many higher-education institutions kept campuses fully or partially closed during the 2020-2021 academic year. Those that remained open relied on a variety of public health measures, including masking, physical distancing, hand sanitizing stations, ventilation adjustments, plexiglass installation, contact tracing teams, quarantine and isolation facilities, and de-densification.^1^ Some institutions also instituted regular population-level surveillance testing to track and contain the spread of SARS-CoV-2 on campuses.^2,3,4^ The resources required to launch and maintain such surveillance programs are likely unrealistic for most organizations.^5^ Nevertheless, the data generated from such approaches can provide valuable insight on strategies for mitigating the spread of SARS-CoV-2.

Many US higher education institutions are now mandating vaccination for returning students,^6^ even as vaccine hesitancy remains vexingly high in the country, with many continuing to question the effectiveness and safety of the vaccines. This study takes advantage of data collected through one university’s surveillance testing and sequencing programs to assess whether there was an association between vaccination coverage and the levels and spread of COVID-19 cases even in the presence of highly transmissible variants and congregate living.

## Methods

In this case series, we report on the spring 2021 semester at a midsized Midwestern university with a total (faculty, staff, and students) population of roughly 16 000, 86% of classes held in-person, and full density in residence halls. The university is located in a midsized town of approximately 100 000 people.

The study population was limited to individuals who belong to the university population (faculty, staff, and students) and were tested for SARS-CoV-2 at least once at the university between January 6 and May 20, 2021. We compared these data with the publicly available county-level data obtained through the state’s public health website. Given that individuals in the university community also belong to the local county, it is likely that the 2 populations overlap to some extent. Given our inability to tease apart these different populations in the county data, the degree of overlap is difficult to assess.

This work followed the reporting guideline for case series. The study was reviewed by the University of Notre Dame’s institutional review board and was classified as exempt for approval and informed consent. Race and ethnicity data were collected by the university as self-reported, but access to those data were restricted to the study team for the analysis reported in this work. The university leadership did not approve the use of that information for this study, and as a result, the data sets provided for analysis did not include race and ethnicity for the university population.

We calculated Pearson correlation coefficients between the percent fully vaccinated and the 7-day moving average of positive cases using RStudio, version 1.4.1717 (R Foundation). The 7-day moving average of positive cases was defined as the average number of positive cases during the last 7 days. Graphical analysis comparing trends in cases with trends in vaccination were developed also using R.

The university developed a comprehensive risk mitigation strategy involving universal entry screening, commercial and in-house surveillance testing, contact tracing, quarantine, isolation, environmental modifications, behavioral policies, and classroom de-densification. As part of this strategy, the university established an in-house Clinical Laboratory Improvement Amendments–certified testing facility with the ability to detect SARS-CoV-2 via reverse transcription-polymerase chain reaction testing and genetically analyze samples for the presence and identity of genetic variants. For the spring semester (January 6 to May 20, 2021, with classes beginning on February 3, 2021), the university substantially increased its general surveillance testing capacity and then required all undergraduates and professional students to be tested once per week (defined as a 7-day period from Monday to Sunday), with voluntary testing occurring every other week for graduate students, faculty, and staff. Additionally, a data-driven adaptive testing program, based on network analysis, was implemented on February 8, 2021, to supplement the general surveillance testing. Individuals in both the general surveillance and adaptive cohorts were notified of their selection for testing via email and text and submitted to either a saliva or nasal reverse transcription-polymerase chain reaction test at the university testing center within 24 hours of notification.

The surveillance testing program relied on both commercial testing (61 256 tests; 916 positives) and in-house reverse transcription-polymerase chain reaction molecular testing (134 929 tests; 687 positives) for SARS-CoV-2 (eMethods in the Supplement). Using the saliva specimens collected in-house, genetic variants of SARS-CoV-2 on 446 samples were determined by next-generation sequencing of the viral genomes with comparison to the extensive databases of known variants.^7,8^

The study population started to become eligible for COVID-19 vaccines via the local public health department on December 14, 2020, with a focus on the older population and health workers. By March 31, everyone over the age of 16 became eligible for the vaccine. To facilitate vaccination of the local and university populations, the university organized several mass vaccination clinics held on March 26 and 27 (local population), April 8 to 15 (university population), and April 29 to May 6 (university population).

## Results

The university conducted 196 185 COVID-19 tests throughout the course of the entire spring 2021 semester and identified 1603 individuals who were positive for SARS-CoV-2, including 1426 students (89.0%), 152 staff (9.5%), 15 faculty (0.9%), and 10 other (0.6%). Of those who tested positive for SARS-CoV-2, 950 were male (59.3%), 644 were female (40.2%), and 1265 were between the ages of 17 and 22 years (78.9%) (Table).

**Table.** Spring Population and Positivity Characteristics

Characteristic	No. (%)	
	Population (n = 14 894)	Positive cases (n = 1594)^a^
Affiliation		
Student	11 091 (74.5)	1426 (89.0)
Staff	2890 (19.4)	152 (9.5)
Faculty	883 (5.9)	15 (0.9)
Other	30 (0.2)	1 (0.1)
Sex^b^		
Female	7005 (47.0)	644 (40.2)
Male	7888 (53.0)	950 (59.3)
Age group, y		
17-22	8692 (58.4)	1265 (78.9)
23-30	2580 (17.3)	170 (10.6)
31-50	2082 (14.0)	102 (6.4)
51-64	1267 (8.5)	52 (3.2)
≥65	273 (1.8)	5 (0.3)

^a^ The total number of positives identified in the study was 1603, which includes 9 individuals who tested positive twice during the course of the study.

^b^ For sex, 1 NA (not applicable) value was reported and is excluded from the table.

**Figure 1.**
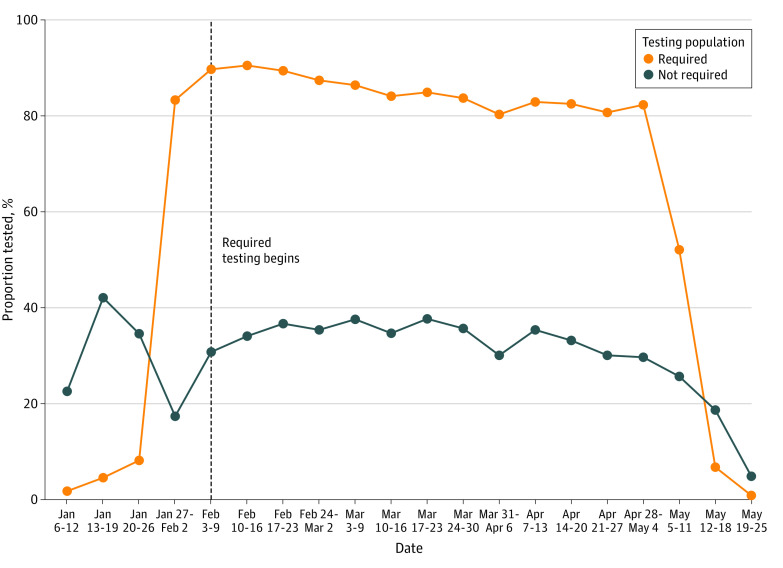
Weekly COVID-19 Testing Percentages of the Required and Voluntary Populations at the University The vertical line on the week of February 3 signifies the start of mandatory testing for undergraduate and professional students.

On any given day during the Spring 2021 semester, 2 cohorts of the population were sampled for surveillance testing. The first, which we refer to as the general surveillance cohort, comprised individuals in the population mandated to test every week. The second, which we refer to as the adaptive cohort, were selected via machine learning models that generate a risk score based on proximity to active cases within the network of the university. Within the general surveillance cohort, required weekly testing for the undergraduate and professional students and voluntary testing for graduate students, faculty, and staff began on February 3. Figure 1 highlights that across the remainder of the semester, the percentage of individuals tested within each group (required and voluntary) remained consistent through April 28. During this period, the mean (SD) group weekly testing compliance rates were 85.2% (3.5%) and 34.3% (2.7%) for the required and voluntary groups, respectively.

**Figure 2.**
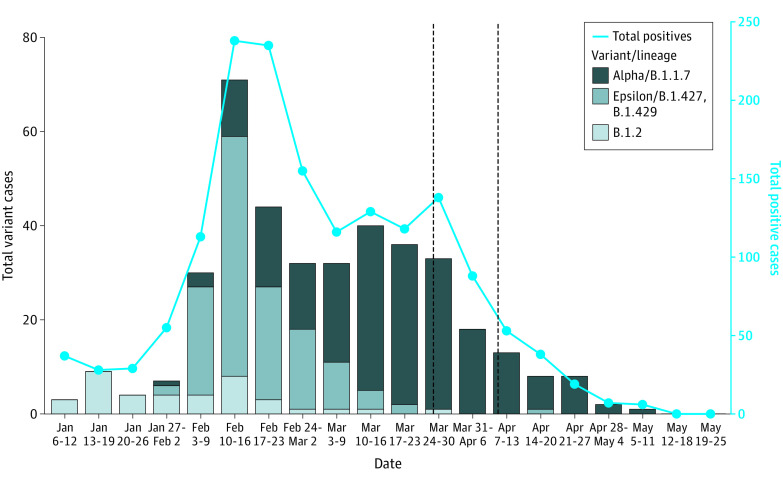
Longitudinal Trend of Positives and Confirmed Variants Seven-day confirmed variant case totals vs total positives are shown. The first vertical dotted line indicates the first date of the vaccination clinic for the local community, March 27, and the second vertical dotted line indicates the first date of the vaccination clinic for the university population, April 8.

Sequenced positive saliva specimens revealed that the only variant present at the start of the semester was the B.1.2 variant. By the end of January, variants designated by the Centers for Disease Control and Prevention as variants of interest and of concern began to appear on campus.^9^ The Epsilon variant quickly rose in frequency to become the dominant variant by mid-February. The Alpha (B.1.1.7) variant was the majority strain within 5 weeks and by 8 weeks after detection accounted for over 90% of cases (Figure 2).

**Figure 3.**
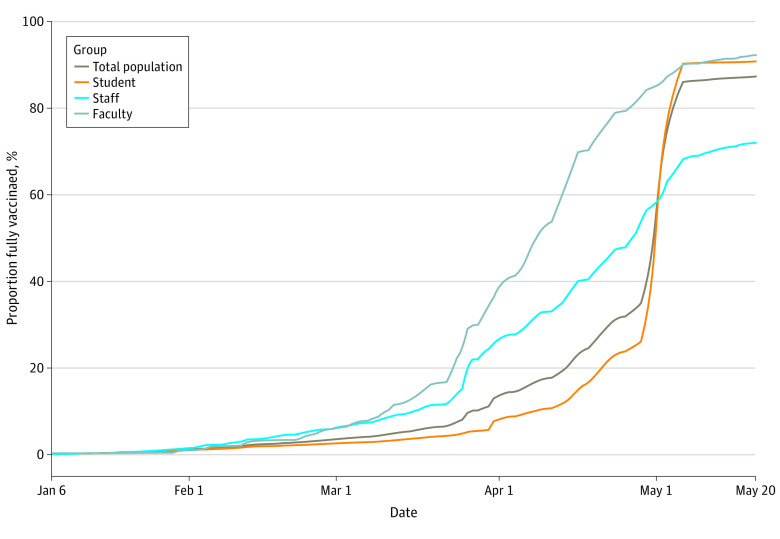
Daily Cumulative Percentage of Fully Vaccinated Individuals by Population Group The brown curve represents the daily cumulative percentage of fully vaccinated individuals of the total university Spring 2021 in-person population; the orange curve represents the rate of vaccination for the university Spring 2021 student population; the blue curve represents the rate of vaccination for the university Spring 2021 staff population; and the gray curve represents the rate of vaccination for the university Spring 2021 faculty population.

**Figure 4.**
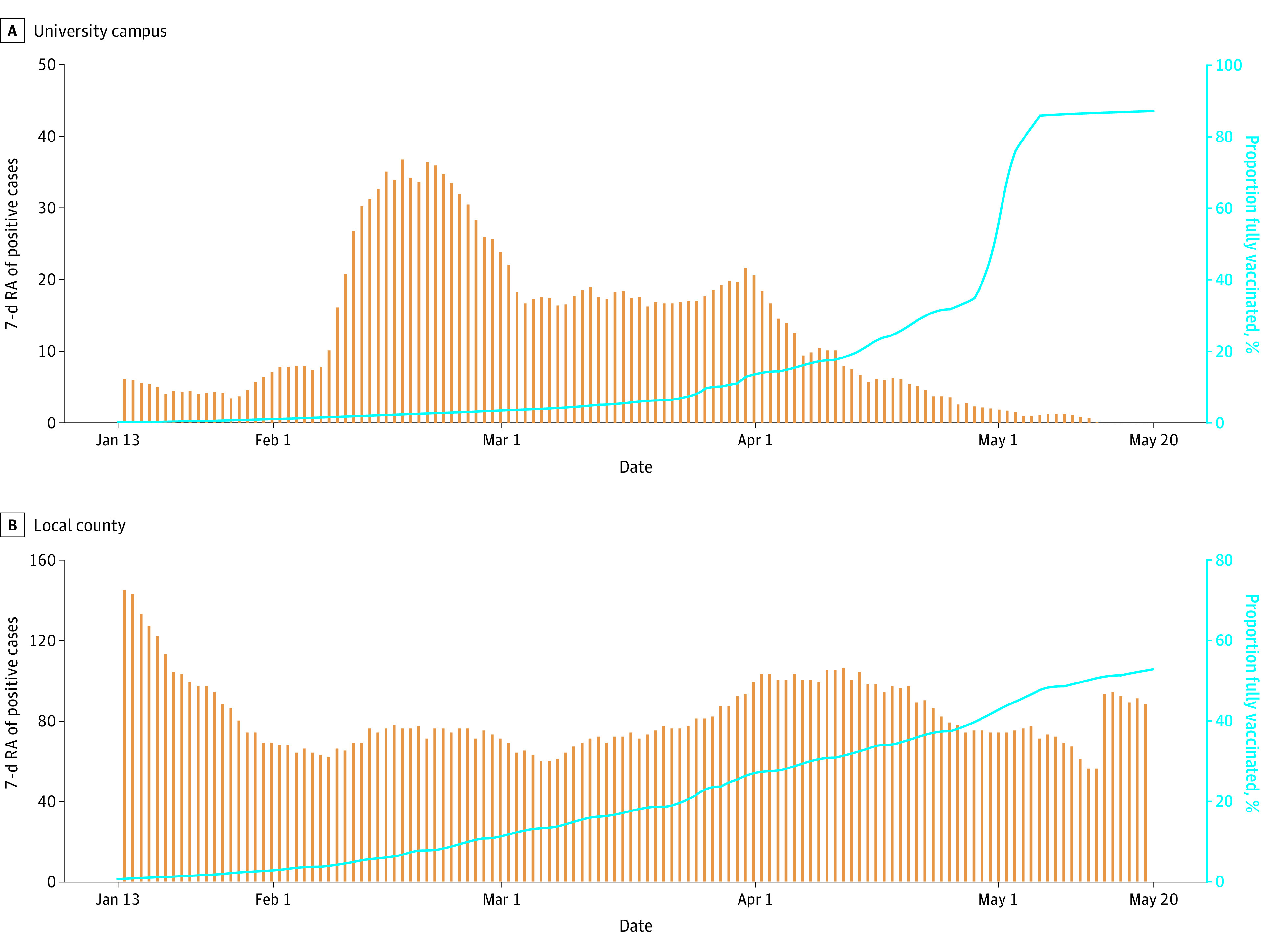
Longitudinal Trend of Positive Cases and Percent Vaccinated in the Population Comparison of (A) university campus and (B) local county 7-day rolling average of positive cases vs the percentage of fully vaccinated population.

From April 8 to 15, the state provided the university with sufficient Pfizer-BioNTech 2-dose vaccines to allow all members of the university community to receive them, including family members of employees, during an on-campus mass vaccination event. The university hosted a second onsite mass vaccination clinic to deliver the second dose of the 2-dose Pfizer-BioNTech vaccine from April 29 to May 6. By May 20, 13 002 of 14 894 individuals from the university community (87.3%) were known to be fully vaccinated, including 10 068 of 11 091 students (90.8%), 814 of 883 faculty (92.2%), and 2081 of 2890 staff (72.0%) (Figure 3). Figure 4 shows the association between percentage of people fully vaccinated and the 7-day moving average of positive cases for both the university and county populations for January through May of 2021. For the university population, we found a Pearson correlation coefficient of −0.57 (95% CI, −0.68 to −0.44), and for the county population, we found a Pearson correlation coefficient of 0.051 (95% CI, −0.22 to 0.12).

## Discussion

For the spring 2021 semester (January 6 to May 20, 2021), the university updated its capabilities to substantially increase its general surveillance testing capacity to require all undergraduates and professional students to be tested once per week (defined as a 7-day period from Monday to Sunday) with voluntary testing occurring every other week for graduate students, faculty, and staff. We note that in-person classes officially began on February 3, with students returning to campus in the preceding week. This accounts for the rise in cases the week of February 3. The wide-scale implementation of surveillance testing isolated positive cases quickly, leading to a decline in cases the week of February 10, with relatively stable case rates thereafter until the vaccination rates began to rise. Additionally, a data-driven adaptive testing program was implemented on February 8, 2021, to supplement the general surveillance testing. The adaptive testing program resulted in higher positivity rates than the general surveillance program and was potentially associated temporally with a decline in cases, indicating its potential usefulness at identifying transmission risk in the population more quickly than the general surveillance approach.^10^

The incidence rate of COVID-19 has been known to be influenced by the type of variant present in the population, with variants of concern being highly-transmissible and quickly increasing the number of positive cases in a population.^11^ In an effort to monitor the impacts of variants of concern in the spread of COVID-19 in the university population, genetic sequencing of positives saliva specimens was conducted.^12^ The next-generation sequencing results revealed that the only variant present at the start of the semester was the B.1.2 variant, and within 5 weeks, the Alpha (B.1.1.7) variant was the majority strain, accounting for more than 90% of cases 8 weeks after detection. The importance of the association we found between vaccination coverage and levels and spread of COVID-19 is strengthened by the fact that the more transmissible Alpha variant was already present and was the leading driver of infections in the community.

Running parallel to the surveillance testing effort at the university was the increased availability of COVID-19 vaccines. The home state of the university began offering vaccines to health care workers and subsequently to the older population on December 14, 2020. By March 31, 2021, eligibility for vaccination opened up to all those over the age of 16 years, with only 15.4% of individuals 18 years or older fully vaccinated in the state by that date.^13^

In an effort to rapidly increase the vaccination rate across the state, local and state governments partnered with local organizations to host mass vaccination clinics. The university hosted a mass vaccination clinic (Johnson & Johnson) for northern state residents on March 26 and 27, which provided ready access to vaccines for all county residents, including university faculty and staff. At this clinic, more than 5000 state residents received vaccines. Figure 3 shows that as a likely result of local and state vaccination efforts, by the start of the university’s mass vaccination campaign on April 8, 50% of the faculty and 30% of the staff were already fully vaccinated.

On April 7, university administration informed the student body that vaccines would be required to enroll during the fall 2021 semester and offered easing of public health restrictions during graduation weekend if the student body achieved at least a 90% vaccination rate. To facilitate this goal, the university, in collaboration with state and local governments, held mass vaccination clinics that offered the Pfizer-BioNTech 2-dose vaccines to all members of the university community (first dose: April 8-15; second dose: April 29-May 6).

Following these mass vaccination clinics, a 90.8% student vaccination rate was achieved by early May, compared with 28% of individuals aged 18 to 24 years fully vaccinated nationwide^14^ and 32% statewide^15^ by the same date. By May 20, 87.3% of the total in-person campus population had been fully vaccinated, compared with 53% of the 18 and older population in the local county (Figure 3).^16^ Given that the university mandated vaccination for students, faculty, and staff with exceptions provided for medical or religious reasons, it is not surprising that vaccine coverage was higher within the university community compared with the local county.

Two weeks after the first dose of the Pfizer BioNtech vaccine, administered between April 21 and 27 (Figure 2), positive cases fell to their lowest levels of the semester, while the same rate of testing was maintained (Figure 1). Positive cases continued to decrease for the remainder of the school year. In the last 2 weeks of the semester, the university did not detect any positive cases.

Figure 4 shows the association between the percentage of people fully vaccinated and the 7-day moving average of positive cases for both the university and the county populations. For the university population, we found that the 7-day moving average of positive cases was inversely associated with the cumulative vaccination rate, with a statistically significant Pearson correlation coefficient of −0.57 (95% CI, −0.68 to −0.44). For the county population, there was no significant association, with a Pearson correlation coefficient of 0.051 (95% CI, −0.22 to 0.12). The higher correlation within the university community likely results from the fact that members of the university community were tested regularly, and even asymptomatic cases (which would be much less likely to be tested in the community) were isolated rapidly. In addition, members of the university mostly interacted with other members of the university community, substantially reducing their exposure to unvaccinated or positive but asymptomatic individuals.

### Limitations

This study has several limitations, including the lack of a statistically valid contemporaneous comparison group. Although we reference trends in the local county and compare them with the university setting, it is difficult to draw comparisons given the vaccine mandates issued by the university as well as the relative insularity of the university community. Additionally, the university general surveillance and adaptive testing was much more comprehensive than what is observed in the county data, making these 2 data sources challenging to compare. The nature of the data also limits our analysis to assessing association rather than establishing causality between vaccine coverage and reduced case numbers. Future research might combine data from universities that undertook similar surveillance testing but different approaches to vaccination as well as explore other statistical methods in an effort to establish a causal relationship between vaccination rates and the spread of COVID-19 cases.

## Conclusions

While weekly surveillance testing as practiced by some colleges and universities to contain the spread of COVID-19 is well beyond the scope of many organizations, data from these efforts are useful for other purposes. This case series found that at a midsized Midwestern university, which had previously exhibited persistent and significant numbers of COVID-19 cases despite robust public health protocols, high vaccination coverage was associated with decreases in numbers of COVID-19 cases within the campus community. This association was seen even with predominantly in-person education, full-density congregate living, and the presence of the more transmissible B.1.1.7 (Alpha) variant. These results support the decision by many colleges and universities to require vaccines and more generally provide evidence of the efficacy of COVID-19 vaccines.

## Notes

Supplement. **eMethods.** Saliva Sample Preparation, RNA Extraction, Illumina Whole Genome Library Preparation and Sequencing, and NGS Data Analysis and Variant Determination
eReferences
